# Exercise, Telomeres, and Cancer: “The Exercise-Telomere Hypothesis”

**DOI:** 10.3389/fphys.2018.01798

**Published:** 2018-12-18

**Authors:** Nikitas N. Nomikos, Pantelis T. Nikolaidis, Caio V. Sousa, Apostolos E. Papalois, Thomas Rosemann, Beat Knechtle

**Affiliations:** ^1^Faculty of Medicine, National and Kapodistrian University of Athens, Athens, Greece; ^2^Exercise Physiology Laboratory, Nikaia, Greece; ^3^Graduate Program in Physical Education, Catholic University of Brasília, Brasília, Brazil; ^4^Experimental Research Center, ELPEN Pharmaceuticals, Attica, Greece; ^5^Institute of Primary Care, University of Zürich, Zürich, Switzerland; ^6^Mebase St. Gallen Am Vadianplatz St. Gallen, Switzerland

**Keywords:** cancer, medicine, exercise, telomere, aging, well-being

## Abstract

Telomeres are genomic complex at the end of chromosomes that protects the DNA and telomere length (TL) is related to several age-related diseases, lifespan, and cancer. On the other hand, cancer is a multifactorial disease that is responsible for reduce the quality of life and kills millions of people every year. Both, shorter TL and cancer are related and could be treated or prevented depending of the lifestyle. In this review we discuss the possible role of exercise in the relationship between shorter telomeres, telomerase activity, and cancer. In summary, there is evidence that exercise leads to less telomere attrition and exercise also may diminish the risk of cancer, these two outcomes are possible intermediated by a reduction in oxidative stress, and chronic inflammation. Although, there is evidence that shorter TL are associated with cancer, the possible mechanisms that one may lead to the other remains to be clarified. We assume that humans under cancer treatment may suffer a great decrease in quality of life, which may increase sedentary behavior and lead to increased telomere attrition. And those humans with already shorter TL likely lived under a poor lifestyle and might have an increased risk to have cancer.

## Introduction

Telomeres are specialized structures at the ends of linear chromosomes (Kipling, 1995; Blackburn et al., 2015). A telomere is a region of repetitive nucleotide (organic molecule) sequences at each termini of a chromosome (DNA molecule), which protects the end of the chromosome to maintain genomic stability, avoiding degradation and fusion (Blackburn, 2010). Telomere’s name is derived from the Greek nouns telos (τ
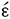
λoς), that means “end” and meros (μ
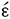
ρoς, root: μερ-), that means “part.” As a physiological cellular process, a small part of telomeric DNA is lost with each cell division (Shammas, 2011). Therefore, telomeres length (TL) shortens every time a cell division occurs, which makes it a marker of biological aging (Blackburn et al., 2015). TL is also associated with large number age-related disorders, such as type 2 diabetes, hypertension, Alzheimer’s and Parkinson’s disease, and even cancer (Shammas, 2011).

When TL is short enough, damage to the DNA may cause the cell to produce non-functional proteins, which may reverberate in two different paths: (i) the non-functional cell is led to apoptosis, which is roughly the senescence process that humans age; or (ii) the non-functional cell continues to produce non-functional proteins, which may become non-functional cell and eventually becomes a cancer (d’Adda di Fagagna, 2008; Maciejowski and de Lange, 2017; Cleal et al., 2018).

On the other hand, life-long exercise practice has being shown to reduce biochemical factors related to telomere attrition, such as oxidative stress (Sousa et al., 2017) and chronic inflammation (Gleeson et al., 2011). There are also some studies reporting that a sedentary behavior is associated with shorter TL (Sjogren et al., 2014), and furthermore, elite athletes, and master athletes have longer TL than their non-athlete counterparts (Muniesa et al., 2017; Simoes et al., 2017).

Although literature is almost consensual pointing the benefits of exercise to prevent and treat a great variety of cancers, the relation between telomere-exercise-cancer still needs clarification. In this narrative review, we aimed to identify the beneficial effects of exercise to prevent and treat cancer. Moreover, we also aimed to identify which methodological aspects are missing in the literature that could help in the development of future experimental designs.

## Telomere and Telomerase Dynamics

Telomeres are specialized functional complexes (parts of DNA molecules) that are cited at the end of DNA; they protect the ends of eukaryotic chromosomes from terminal attrition (Blackburn, 2001). On the other hand, telomerase is an enzyme responsible for synthesizes DNA at the chromosome terminal, lengthening the terminal regions of telomeric DNA by RNA-templated addition of tandemly repeated (Blackburn, 2001).

Telomerase is an RNA-dependent DNA polymerase (ribonucleoprotein) that synthesizes telomeric DNA sequences and provides the molecular basis for unlimited proliferative potential (Cong et al., 2002). Furthermore it counteracts telomere erosion and under certain circumstances may also elongate TL (Aviv, 2002). Telomerases include a protein subgroup of specialized reverse transcriptase enzymes known as Telomerase Reverse Transcriptase (TERT). The modulation of telomerase activity may have significant anti-aging implications as well as anticancer therapy (Cong et al., 2002). A study previously reported that nucleotide polymorphisms in the TERT, are strongly associated with decreased breast cancer risk (Helbig et al., 2017).

## Telomeres and Aging

Telomeres are the parts of chromosomes that affect aging, and their length (TL) appears to be an indicator of biological age (Sanders and Newman, 2013; Zhang et al., 2014; Jylhava et al., 2017). Telomeres become shorter due to cell division and lifestyle factors; critically shortened telomeres are linked to cellular dysfunction and aging (Garcia-Martin et al., 2017). Telomeres shorten with age, with each round of mitosis (division of the nucleus), due to the inability of the DNA replication mechanisms to read and copy the ends of linear chromosomes (phenomenon known as the “end replication problem”), and also from oxidative stress, which induces DNA damage (Eisenberg, 2011).

Telomeres act as a buffer against the loss of coding or regulatory DNA during cell proliferation (Helbig et al., 2017). Regarding the role of telomerase activity on cell aging, it has being shown that cells with a perfect regulated telomerase activity are considered immortal because they can divide past the Hayflick limit without entering in phase of senescence (biological aging) or apoptosis.

Shorter TL is also closely associated to almost all age-related diseases (Blackburn et al., 2015), and the literature has been slashing to point that the main reason modulating a possible acceleration or attenuation in this process is the lifestyle (Blackburn et al., 2015; Denham et al., 2016). An inadequate lifestyle and aging are both closely related to chronic inflammation (Gleeson et al., 2011), increased oxidative stress (Sousa et al., 2017) and decrease in telomerase activity (Blackburn et al., 2015).

## Telomeres and Exercise

Lifestyle is a multifactor aspect with regards to general health and could be simplified as nutrition habits, stress management, and physical activity/sedentary behavior. In one of the first articles on this topic, Puterman et al. (2010) reported that exercise might have a great effect on attenuating telomere attrition. In a larger study, Sjogren et al. (2014) showed that avoiding sedentary behavior might also attenuate telomere attrition.

It seems that avoiding sedentary behavior leads to well-being, but adding exercise daily may lead to even better well-being in terms of telomere preservations. Elite athletes have longer TL than their counterparts (Muniesa et al., 2017) and master athletes (around 40–50 years old) also have longer TL than age-matched non-athletes (Østhus et al., 2012; Simoes et al., 2017).

The mechanisms by which exercise could attenuate cell senescence and preserve TL have being studied especially in animal models (Werner et al., 2009; Cunha et al., 2018). Werner et al. (2009) reported that increased levels of PA are associated with elevated telomerase activity and suppression of several apoptosis proteins, such as p53 and p16. On the other hand, Cunha et al. (2018) showed that more vigorous exercise (above lactate threshold) could also diminish p53 activity, but also decrease the expression of proteins related to telomere protection (sheltering proteins). These authors suggest that this may happen because in a more balanced environment, there is no need to a larger expression of sheltering proteins.

In summary, the body of available evidence shows that one of the factors that exercise may lead to an attenuated telomere attrition and aging is the maintenance of good body composition (Sousa-Victor et al., 2015; Simoes et al., 2017). Which, in turn, it likely helps to maintain a good metabolic balance, as well as healthy states of oxidative stress and inflammatory status (von Zglinicki, 2002).

## The Interplay

Oxidative stress seems to play a central role in which TL could increase the cancer risk. The Long Island Breast Cancer Study Project (Gammon et al., 2002) explored the relationship between TL and breast cancer risk, examining whether dietary intake of antioxidants would have any effect. The authors of this project reported that a moderate increase in breast cancer risk was observed among women with the shortest telomeres and with lower dietary and supplemental intake of beta-carotene, vitamin C or E. These results provided the evidence that breast cancer risk may be affected by TL among premenopausal women or women with low dietary intake of antioxidants or in an unbalanced oxidative stress status (Shen et al., 2010). Antioxidants dietary intake (through supplementation or general food) is important to a healthier redox status and TL maintenance (Noorimofrad and Ebrahim, 2018). However, the regulation of endogenous antioxidant capacity by regular exercise has led to favorable results (Radak et al., 2008; Sousa et al., 2017).

In that context, exercise seems to play a central role in cancer treatment and prevention, and also in TL maintenance. Nevertheless, the available literature shows that this may occur by different pathways. Biologically, longer TL means that a cell is young and has not been divided too many times yet. Roughly, cell division happens when another cell is needed (Allsopp et al., 1995). If the need for new cells is greater than the capacity of cell to multiply themselves, the tissue starts to lose their function little by little, that is aging (Allsopp et al., 1995). However, cancer cell function as the opposite of that, this mutant genes have a high division rate and normally have longer TL and telomerase activity (Macieira-Coelho, 1994; Brinkley, 2001).

Rapidly dividing cells causes cancer, and some of those (i.e., esophageal and ovarian) are associated with shorten TL (Risques et al., 2007; Wentzensen et al., 2011). McGrath et al. (2007) observed, in both men and women, that those with bladder cancer had shorter TL than controls. They also evaluated the effect of smoking on TL and found significantly shorter telomeres in healthy individuals who smoked than in those who did not (McGrath et al., 2007). A series of epidemiological studies found that having shorter overall TL from blood leucocytes was significantly associated with an increased risk of bladder, head and neck, lung, and renal cell cancers (Zheng et al., 2010).

Pooley et al. (2010) found a strong association between shorter telomere length and breast cancer status in the retrospective study, with an OR of ∼15 for women in the bottom quartile of TL compared to the top quartile (Pooley et al., 2010). They also stated that the prospective breast cancer study showed evidence of an association, but the effect was not statistically significant and the majority of telomere attrition occurred after cancer diagnosis rather than before or during cancer (Pooley et al., 2010). Martinez-Delgado et al. (2012) reported that shorter telomere length is associated with increased ovarian cancer risk in familial and sporadic cases (Martinez-Delgado et al., 2012). Patients with colorectal cancer (CRC) displayed clear evidence of telomere attrition compared with controls (Maxwell et al., 2011). Furthermore telomere length could be a non-invasive blood biomarker to pre-screen for risk of advanced adenomas (AAs); PBL telomeres are shortened in patients with colorectal neoplasia (Riegert-Johnson et al., 2012). Short TL and subsequent genomic instability contribute to malignant transformation, and it is therefore contingent that people with minor TL are at higher risk for different types of cancer (Martinez-Delgado et al., 2012).

Furthermore, Ma et al. (2011) performed a meta-analysis and reported that, although shortened TL are associated with increase risk of bladder and lung cancer, this association was not found for breast cancer. On the other hand, another study showed that an increased breast cancer mortality following diagnosis to women with elevated telomere shortening (Duggan et al., 2014). More recently, Sanft et al. (2018) performed a randomized controlled trial in women with breast cancer, from the total sample size (*n* = 151), 93 were submitted to a lifestyle intervention that included weight loss strategies with exercises and nutrition and 58 to usual care. The authors reported that, the lifestyle intervention in breast cancer may lead to TL maintenance, but the effect may be limited to patients in the earlier stages (0/I) of breast cancer.

**FIGURE 1:**
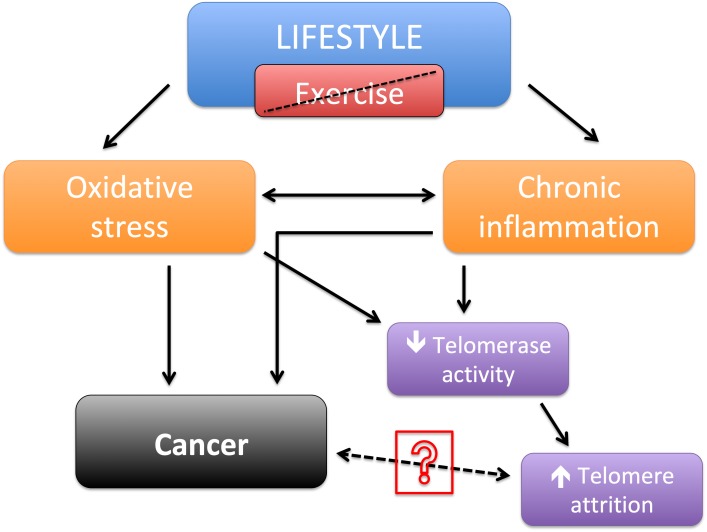
Schematic of lifestyle without exercise leading to shortened telomere length and/or cancer.

Taken separately, in a meta-analysis was showed evidence that perceived psychological stress is associated with a decrease in TL (Mathur et al., 2016). In several other observational studies, was evidenced that exercise as could reduce telomere attrition and contribute to their length maintenance (Kim et al., 2012; Ornish et al., 2013; Puterman et al., 2018). And also that most types of cancer, although are closely related to telomere, they are also strongly associated with lifestyle (Eisenberg, 2011; Kerr et al., 2017). Therefore, it is reasonable to suggest that shorter telomere length may not be a direct cause for cancer, but a result of inadequate lifestyle choices that possibly led to both increased risk of cancer and shorter TL (Figure 1). The possible pathway between cancer and TL still needs clarification.

## Conclusion

In summary, there is evidence that exercise leads to less telomere attrition and exercise also may diminish the risk of cancer, these two outcomes are possible intermediated by a reduction in oxidative stress and chronic inflammation. Although, there is evidence that shorter TL are associated with cancer, the possible mechanisms that one may lead to the other remains to be clarified. We assume that humans under cancer treatment may suffer a great decrease in quality of life, which may increase sedentary behavior and lead to increased telomere attrition. And those humans with already shorter TL likely lived under a poor lifestyle and might have an increased risk to have cancer.

## Author Contributions

NN designed the review and collected the data. All authors contributed by writing, editing, and reviewing the manuscript, and read and approved the final manuscript.

## Conflict of Interest Statement

The authors declare that the submitted work was not carried out in the presence of any personal, professional, or financial relationships that could potentially be construed as a conflict of interest.
